# Silage making of maize stover and banana pseudostem under South Ethiopian conditions: evolution of pH, dry matter and microbiological profile

**DOI:** 10.1111/1751-7915.13626

**Published:** 2020-07-24

**Authors:** Ashenafi Azage Mitiku, Addisu Fekadu Andeta, An Borremans, Bart Lievens, Sofie Bossaert, Sam Crauwels, Ben Aernouts, Yisehak Kechero, Leen Van Campenhout

**Affiliations:** ^1^ Department of Microbial and Molecular Systems Lab4Food KU Leuven Geel Campus Geel Belgium; ^2^ Leuven Food Science and Nutrition Research Centre (LFoRCe) KU Leuven Leuven Belgium; ^3^ Department of Animal Sciences College of Agricultural Sciences Arba Minch University Arba Minch Ethiopia; ^4^ Department of Biology College of Natural Sciences Arba Minch University Arba Minch Ethiopia; ^5^ Laboratory for Process Microbial Ecology and Bioinspirational Management (PME and BIM) Department of Microbial and Molecular Systems KU Leuven De Nayer Campus Sint‐Katelijne Waver Belgium; ^6^ Department of Biosystems Livestock Technology KU Leuven Geel Campus Geel Belgium

## Abstract

The study was conducted to evaluate the microbial dynamics during silage of maize stover and banana pseudostem in the environmental conditions of southern Ethiopia. To meet this objective, microsilos containing either maize stover or banana pseudostem, both with and without molasses, were prepared. Subsequently, samples were analysed on day 0, 7, 14, 30, 60 and 90 of the fermentation process. As a result, on day 7, all treatments except banana pseudostem without molasses showed a significant reduction in pH. It was also this silage type that supported the growth of Enterobacteriaceae longer than three other silage types, i.e. until 30 days. The yeasts and moulds and the *Clostridum* endospore counts also showed a reducing trend in early fermentation and afterwards remained constant until day 90. Illumina MiSeq sequencing revealed that *Leuconostoc, Buttiauxella* species and Enterobacteriaceae were the most abundant bacteria in the initial phases of the fermentation. Later on, *Buttiauxella*, *Lactobacillus*, *Weissella* and *Bifidobacterium* species were found to be dominant. In conclusion, silage of the two crop by‐products is possible under South Ethiopian conditions. For banana pseudostem, the addition of molasses is crucial for a fast fermentation, in contrast to maize. Upscaling needs to be investigated for the two by‐products.

## Graphical abstract

Maize and Banana pseudostem by‐products can be considered as potential options in silage making under the environmental conditions of Ethiopia. Fermentation of banana pseudostem requires molasses to obtain a fast pH reduction.

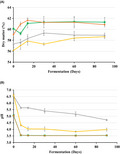

## Introduction

Tropical countries typically suffer from seasonal variations of available feed, both in quantity and quality (Salo, 2017). As a result, poor quality natural pastures and crop residues are the major components of today’s livestock feed in Ethiopia (Kechero *et al*., 2014). A better use of locally available feed sources, which cannot be applied as human foods, is therefore getting more and more attention (Moselhy *et al*., 2015). In this regard, preservation of agricultural by‐products, such as banana pseudostem and maize stover, may contribute to secure livestock feed. The banana plant is a herbaceous perennial species of the Musa family, grown widely as a fruit crop in both tropical and subtropical regions (Wang *et al*., 2016). About 69% (± 37 thousand ha) of the Ethiopian land coverage for banana production is situated in the southern and south‐western parts of the country, accounting for 77.53% (± 370 thousand tonnes) of the Ethiopian annual fresh banana production by 22.38% (± 1.5 million) of the banana producers (Alemu, 2017). The Gamo Gofa Zone is also known as a maize growing belt of the southern region covering about 51 442 ha of land and producing 189 655 tons of maize per annum (CSA, 2016).

Currently, smallholder farmers are underutilizing the by‐products of these two crops. The maize is delayed for drying and leaf shattering until the grain is harvested. The banana pseudostem is left aside in the field to decompose and to be used as organic fertilizer, but as there is an excess of banana biomass, the decomposition in the field can cause environmental pollution (Li *et al*., 2010; Yousef *et al*., 2019). Fermentation of forage is a lifelong tradition to conserve feed for periods of shortage caused by inadequate pasture and climate conditions (McEniry *et al*., 2006; Romero *et al*., 2015; Gharechahi *et al*., 2017). During silage, sugars are metabolized into organic acids by lactic acid bacteria (LAB) allowing storage for a long time while maintaining the nutritional quality and improving the palatability of the feed (Storm *et al*., 2010; Moselhy *et al*., 2015; Romero *et al*., 2015; Wang *et al*., 2016; Gharechahi *et al*., 2017; Ni *et al*., 2018). Several studies have been conducted on ensiling whole maize silage in different parts of the world (Israelsen *et al*., 2009; Storm *et al*., 2010), but not yet under tropical conditions as in South Ethiopia. Moreover, ensiling the whole maize plant including the fruits for feed purposes inhibits the use of the grain kernels for human use and is not appropriate for developing countries such as Ethiopia (Israelsen *et al*., 2009). In South Ethiopia, the spikes with grains are removed for human consumption and the crop is harvested relatively late. These two practices can be hypothesized to reduce the soluble carbohydrate content (WSC) available for microbial fermentation. Therefore, addition of molasses can substitute the WSC and enhance lactic acid fermentation (McDonald *et al*., 1991; Ni *et al*., 2018; Liu *et al*., 2020; Shiau *et al*., 2020). To the best of our knowledge, there is only one study in China (Wang *et al*., 2016) reporting on the feasibility of ensiling banana pseudostem for feed purposes, and bacterial profiles have not yet been determined by Illumina sequencing.

In this regard, the aim of this study was to evaluate the microbiological aspects of maize stover and banana pseudostem while ensiling under the environmental conditions prevailing in South Ethiopia, as a first step in judging the feasibility of the process to contribute to feed security. To this end, small‐scale fermentations were performed with the two crops, while monitoring pH, dry matter changes and microbiological profile, using both culture‐dependent (classic microbial counts) and culture‐independent (metagenetic analysis) methods.

## Results and discussion

### Dry matter content and pH

**Fig. 1:**
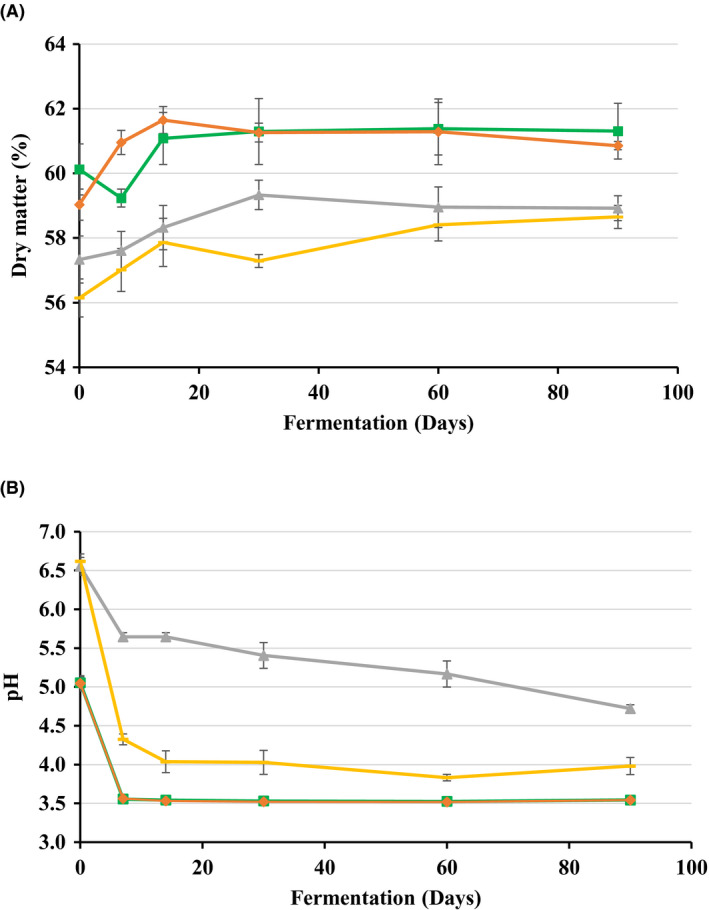
Changes in dry matter content (A) and pH (B) of maize stover without and with molasses (Mwo and Mw) and banana pseudostem without and with molasses (BPSwo and BPSw) during fermentation in microsilos. For pH, the curve of Mwo lies completely behind with that of Mw and it is therefore not visible on the figure.

The dry matter (DM) content at the start and during the four types of fermentation can be seen in Figure 1A. According to Romero *et al*. (2015), the silage evaluated in this study can be classified as low moisture silage having 54–64% DM content. The fresh, unsqueezed BPS exhibited a very low DM content about 12%, which is in agreement with the findings of Wang *et al*. (2016). Because a too high moisture content (or forage having less than 20% DM content) involves the risk of growth of spoilage microorganisms such as *Clostridium* species (McEniry *et al*., 2007; König *et al*., 2017), the chopped BPS was manually squeezed to increase the DM content. As can be seen in Figure 1A, there was statistically significant (*P* = 0.007) difference in the DM contents of the forages used at the start of ensiling. These variations can be due to differences between the two crops and also to the addition of molasses (Ni *et al*., 2017). There was also a statistically significant increase (*P* = 0.012, and *P* = 0.000) in DM content from day 0 to day 90 in all treatments. DM loss from volatile products that may have escaped during oven drying was not taken into account, however. The DM content increased much faster in the first 14 days compared to the rest of the fermentation period.

The pH of the biomass (Fig. 1B) recorded prior to ensiling showed significant differences (*P* = 0.000) between the two crop by‐products. The variation in pH was likely due to differences in composition of the materials used in the experiment. At day 7, all treatments except BPSwo showed a significant decrease (*P* = 0.000) in pH. On day 90, there was a statistically significant difference (*P* = 0.000) in pH between maize, BPS with molasses and BPS without molasses. Moreover, no significant difference was found in maize with or without adding molasses. The maize stover fermentations displayed a rapid reduction in pH to values below 4 on day 7, regardless of molasses being present or not, after which it remained fairly constant. The reduction in pH was comparable with that observed by Gharechahi *et al*. (2017), who also studied the microbial dynamics of maize silage (under Iranian conditions). A significant decrease in pH was also observed for BPSw from day 0 to day 7 to a value of about 4.32 ± 0.07, and the decrease steadily continued to its lowest value reaching a value of 3.83 ± 0.04 on day 60 and tended to increase to 3.98 ± 0.11 on day 90. A comparable drop in pH was also reported by Wang *et al*. (2016) on the fermentation of banana pseudostem without squeezing (9.77 ± 0.16 dry matter content) and without adding molasses. Unlike the other three treatments, the pH of the BPSwo silages remained above 4.5 during the 90 days of ensiling. The significant difference (*P* = 0.000) between the two banana‐based silages can only be related to the addition of the molasses that might have replaced the lost water soluble carbohydrates during squeezing (Ni *et al*., 2017; Bernardes *et al*., 2018). While some fungi and yeasts can grow at low pH values, a rapid decrease in pH to a value below 4.5 likely contributes to the inhibition of spoilage and pathogenic microorganisms (Hu *et al*., 2018; Liu *et al*., 2020).

### Microbial counts

Microbial counts measured during ensiling are shown in Table 1. On day 0, the TVC did not differ significantly (*P* = 0.815) among treatments. On day 7, only the Mwo, Mw and BPSw treatments had reached a maximum in the TVC when considering the whole silage period, while the BPSwo reached a maximum on day 14. Between day 0 and day 90, all TVC decreased with 2.17, 2.75, 1.92 and 1.34 log cfu g^−1^ for Mwo, Mw, BPSwo and BPSw, respectively, which can be explained by a depletion of nutrients for growth and/or a decreasing pH.

**Table 1:** Microbial counts of maize stover and banana pseudostem without and with molasses additive.

Treatments	Microbial group	Microbial counts (log cfu g^−1^)					
		Day 0	Day 7	Day 14	Day 30	Day 60	Day 90
Maize without molasses	Total viable aerobic counts	7.82 ± 0.29^bA^	9.33 ± 0.43^aA^	8.28 ± 0.15^bA^	7.65 ± 0.06^bC^	6.41 ± 0.27^cB^	5.65 ± 0.22^dB^
	Lactic acid bacteria	6.38 ± 0.13^dB^	9.05 ± 0.18^aB^	8.27 ± 0.11^bAB^	7.59 ± 0.16^cB^	6.38 ± 0.17^dC^	5.70 ± 0.21^eB^
	Enterobacteriaceae	6.75 ± 0.19^aB^	< 1.00 ± 0.00^bB^	< 1.00 ± 0.00^bB^	< 1.00 ± 0.00	< 1.00 ± 0.00	< 1.00 ± 0.00
	Yeasts and moulds	6.30 ± 0.25^aA^	4.56 ± 0.08^bB^	4.59 ± 0.09^bB^	4.56 ± 0.05^bA^	4.45 ± 0.14^bA^	4.26 ± 0.29^bA^
	*Clostridium* spores	3.77 ± 0.01^aB^	2.83 ± 0.14^bA^	2.58 ± 0.17^bA^	2.40 ± 0.06^bB^	2.49 ± 0.04^bA^	2.85 ± 0.58^bA^
Maize with molasses	Total viable aerobic counts	7.81 ± 0.16^bcA^	9.50 ± 0.12^aA^	8.49 ± 0.36^bA^	7.52 ± 0.02^cD^	6.37 + 0.32^dB^	5.06 ± 0.23^eC^
	Lactic acid bacteria	6.84 ± 0.22^dAB^	9.38 ± 0.03^aA^	8.05 ± 0.14^bAB^	7.51 ± 0.05^cB^	6.30 + 0.19^eC^	5.06 ± 0.13^fC^
	Enterobacteriaceae	6.76 ± 0.21^aB^	< 1.00 ± 0.00^bB^	< 1.00 ± 0.00^bB^	< 1.00 ± 0.00	< 1.00 ± 0.00	< 1.00 ± 0.00
	Yeasts and moulds	6.33 ± 0.16^aA^	4.68 ± 0.17^bcB^	4.94 ± 0.23^bAB^	4.69 ± 0.19^bcA^	4.23 + 0.16^cA^	4.24 ± 0.35^cA^
	*Clostridium* spores	3.93 ± 0.02^aA^	2.87 ± 0.11^bA^	2.34 ± 0.23^cA^	2.39 ± 0.04^cB^	2.67 + 0.07^bcA^	3.02 ± 0.29^bA^
Banana pseudostem without molasses	Total viable aerobic counts	7.69 ± 0.12^bA^	8.33 ± 0.06^aB^	8.54 ± 0.09^aA^	7.80 ± 0.06^bB^	7.03 ± 0.26^cAB^	5.77 ± 0.27^dB^
	Lactic acid bacteria	6.93 ± 0.16^bA^	8.24 ± 0.13^aC^	7.75 ± 0.47^aB^	6.92 ± 0.05^bC^	6.98 ± 0.25^bB^	5.75 ± 0.24^cB^
	Enterobacteriaceae	7.51 ± 0.08^aA^	4.29 ± 0.19^bA^	3.71 ± 0.23^cA^	< 1.00 ± 0.00	< 1.00 ± 0.00	< 1.00 ± 0.00
	Yeasts and moulds	6.52 ± 0.03^aA^	6.29 ± 0.05^aA^	5.42 ± 0.13^bA^	4.76 ± 0.09^cA^	4.61 ± 0.20^cA^	4.67 ± 0.09^cA^
	*Clostridium* spores	3.02 ± 0.03^aC^	2.88 ± 0.04^abA^	2.62 ± 0.05^cA^	2.63 ± 0.07^bcA^	2.84 ± 0.12^abcA^	2.72 ± 0.17^bcA^
Banana pseoudostem with molasses	Total viable aerobic counts	7.89 ± 0.20^cA^	8.90 ± 0.10^aAB^	8.75 ± 0.24^abA^	8.24 ± 0.04^bcA^	7.84 ± 0.41^cA^	6.55 ± 0.08^dA^
	Lactic acid bacteria	6.91 ± 0.26^dA^	8.92 ± 0.07^aB^	8.65 ± 0.35^abA^	8.22 ± 0.09^bA^	7.62 ± 0.19^cA^	6.65 ± 0.11^dA^
	Enterobacteriaceae	7.50 ± 0.02^aA^	< 1.00 ± 0.00^bB^	< 1.00 ± 0.00^bB^	< 1.00 ± 0.00	< 1.00 ± 0.00	< 1.00 ± 0.00
	Yeasts and moulds	6.29 ± 0.29^aA^	6.22 ± 0.05^aA^	4.81 ± 0.29^bB^	4.59 ± 0.34^bA^	4.50 ± 0.20^bA^	4.59 ± 0.11^bA^
	*Clostridium* spores	3.01 ± 0.00^aC^	2.67 ± 0.02^abA^	2.62 ± 0.01^abA^	2.57 ± 0.07^bA^	2.68 ± 0.33^abA^	2.74 ± 0.15^abA^

Data are the mean of three samples, one from each microsilo (three per treatment) ± standard deviation. Data among sampling days and sample types were analysed using one‐way analysis of variance (ANOVA). The statistical differences among means having significant difference were determined using *Turkey’s* multiple comparisons. Differences were considering significance at *P* < 0.05.

^a,b,c,d,e,f^Different superscripts within the same row indicate significant differences (*P* < 0.05).

^A,B,C^Different superscripts per type of microbial count within the same column indicate significant differences (*P* < 0.05).

Ensiling is based on the available nutrients, such as soluble carbohydrates, and the naturally occurring LAB on the leaves and stems of forages (Wang *et al*., 2016). LAB are responsible for the preservation of fermented forages through production of organic acids, mainly lactic acid and acetic acid (Brusetti *et al*., 2006; Storm *et al*., 2010). Even though statistical differences were noted between the treatments at day 0, from a microbiological perspective, the biomass contained about the same density of LAB in all four types of silages ranging between 6.38 and 6.93 log cfu g^−1^. A maximum in the LAB count was obtained at day 7 for all treatments, and also for all treatments, the counts decreased from then on to result in the lowest values (and for all treatments significantly lower than the value at day 0) at day 90. Interestingly, BPSwo attained the lowest TVC of all four treatments at days 7, 14 and 30, albeit not always statistically. It is plausible that the growth of LAB being the least pronounced for BPSwo is caused by the expected loss of nutrients (not analysed in the current study) due to the squeezing. This could reduce the lactic acid production and explain the diminished pH reduction among all treatments (Dunière *et al*., 2013).

A further consequence of this is likely to be seen in the dynamics of the Enterobacteriaceae. A first observation for this bacterial subgroup is that on day 0, the two banana‐based treatments contained a statistically (*P* = 0.000) higher Enterobacteriaceae count than the two maize‐based treatments. For all treatments, except for BPSwo, the number of Enterobacteriaceae was below the detection limit of 1.00 log cfu g^−1^ as from day 7. In the case of BPSwo, Enterobacteriaceae were only below the detection limit as from day 30, probably due to the fact that pH was not so low and LAB counts not so high as in the other three treatments. Therefore, the squeezing of banana pseudostem without addition of molasses may hinder the initiation and dominance of beneficial microorganisms within the recommended 7–10 days of ensiling (Borreani *et al*., 2018).

On day 0, the yeast and mould counts showed no statistically significant differences among treatments. On day 7, there was statistically significant (*P* = 0.000) reduction in the yeast and mould counts in the Mwo and Mw treatments. A reduction was also observed in BPSwo and BPSw treatments, but it appeared later, i.e. at day 14. At the end of ensiling, the yeast and mould counts did not show a significant difference (*P* = 0.117) between the treatments and the average counts per treatment were between 4.24 and 4.67 log cfu g^−1^. Yeasts and moulds in general can grow at a lower pH than bacteria, but the reduction in the pH occurring in the fermentations in our study, possibly combined with a depletion of available nutrients, caused a general decline in the counts for all treatments. Although no clear relation exists between fungal growth and mycotoxin production, high fungal counts are to be avoided in order to limit the risk of contamination with mycotoxins and other deleterious effects, such as nutrient use and taste changes in the ensiled crop (Wambacq *et al*., 2013).

Finally, all treatments contained *Clostridium* spores at the start of silage were above the limit of detection 2 log cfu g^−1^ (König *et al*., 2017). The higher count seen on maize compared to banana pseudostem might relate to the soil contamination present on the former, whereas for banana pseudostem, the outer layers were discarded (Dunière *et al*., 2013). While during the first weeks of silage, all treatments exhibited a reduction in the number of *Clostridium* spores, they all revealed a slight increase during the last two months of the experiment, yielding values that did not statistically differ among treatments (*P* = 0.699). Attention should be paid to the slight increase in *Clostridium* spores, because when pH remains above 4.5, as in the case of BPSwo, there is a risk of secondary fermentation by *Clostridium* of lactic acid into for instance acetic and unpalatable butyric acid and of degradation of proteins and amino acids (Wilkinson, 2005). Growth of *Clostridium* is unlikely in forages having a DM content of greater than 40% (König *et al*., 2017). Such forages used for silage making are characterized by a high osmotic pressure and a reduced dissociation of organic acids produced during fermentation to keep the pH low (Silva *et al*., 2015; König *et al*., 2017). Therefore, the squeezing activity performed on the chopped BPS resulted in an increase in DM content from 12% to about 56%, thereby reducing *Clostridium* growth and sporulation, even at the higher pH in BPSwo (König *et al*., 2017). Generally, silages containing *Clostridium* spore counts of more than 2 log cfu g^−1^, a high pH (i.e. above 4.5), a low DM content (i.e. below 20%), substantial Enterobacteriaceae counts and an unpleasant smell can be considered as bad silages that may negatively affect the performance and health of livestock (McEniry *et al*., 2006; Wambacq *et al*., 2013).

### Bacterial community composition

**Fig. 2:**
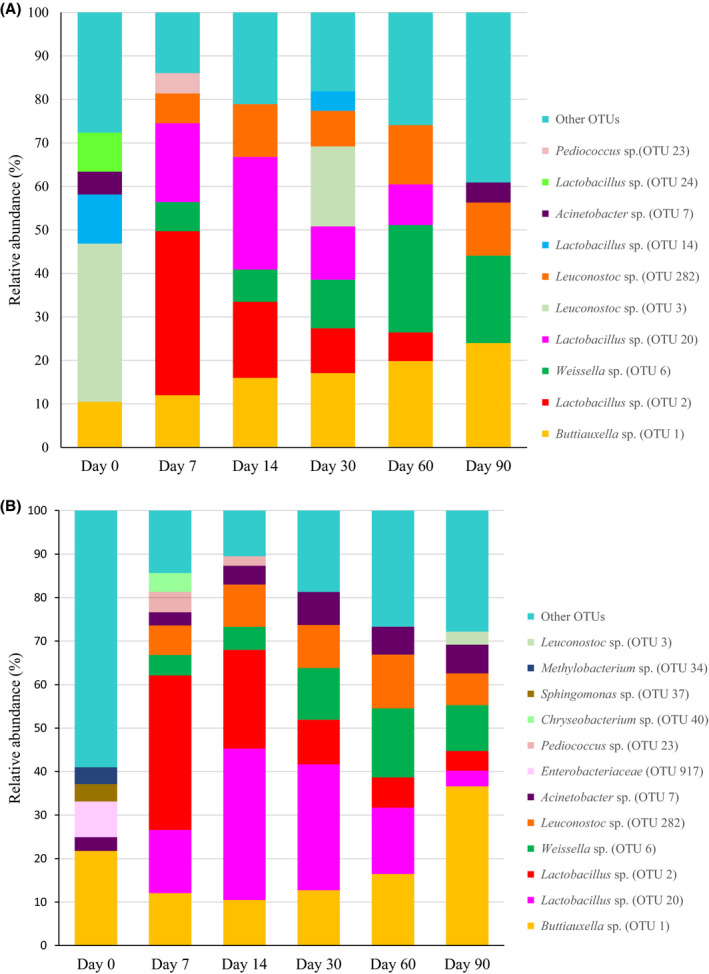
Relative abundances (%) of the bacterial community composition of maize without molasses (A) and with molasses (B) during fermentation in microsilos. OTUs with a relative abundance below 3% are grouped together in ‘Other OTUs’.

**Fig. 3:**
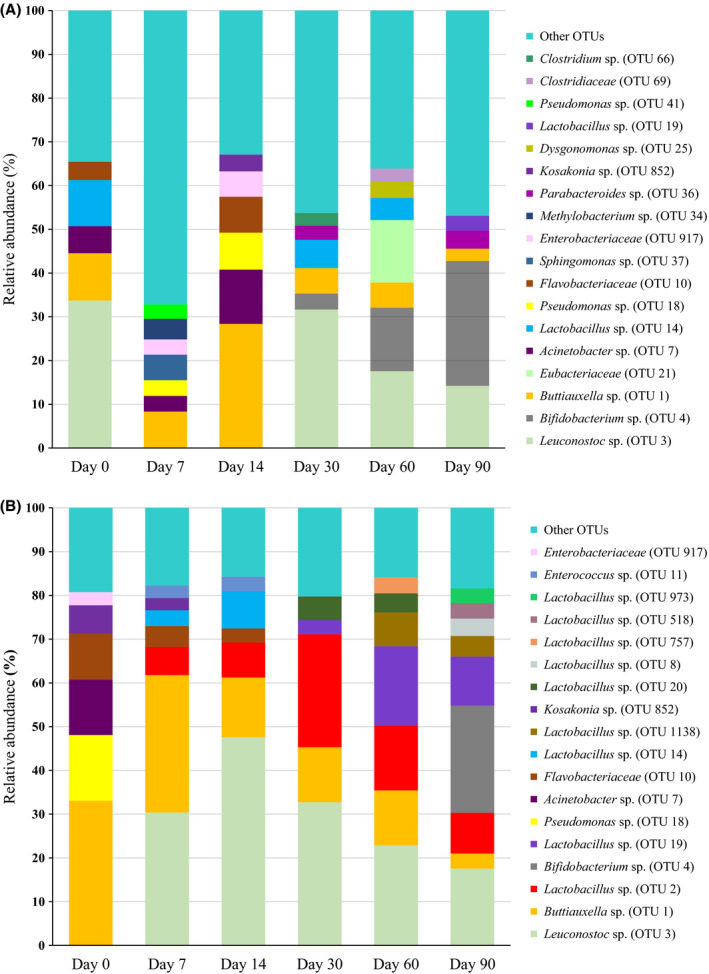
Relative abundances (%) of the bacterial community composition of banana pseudostem without molasses (A) and with molasses (B) during fermentation in microsilos. OTUs with a relative abundance below 3% are grouped together in ‘Other OTUs’.

The bacterial profiles per treatment during fermentation as determined by metagenomics are presented in Figures 2 and 3 as operational taxonomic units (OTUs). The most important similarities in the bacterial community of the four treatments on day 0 were the presence of a *Buttiauxella* sp. (OTU 1; 10.50%, 21.76%, 10.82% and 33.04% relative abundance for Mwo, Mw, BPSwo and BPSw respectively) and an *Acinetobacter* sp. (OTU 7; 5.24%, 3.17%, 6.20% and 12.6% for Mwo, Mw, BPSwo and BPSw respectively). *Leuconostoc* sp. (OTU 3; 36.34% and 33.71%) and *Lactobacillus* sp. (OTU 14; 11.3% and 10.55%) were only found on Mwo and BPSwo, respectively, on day 0. In addition, another *Lactobacillus* sp. (OTU 24; 8.98%) was only found in the Mwo silage type at day 0. Enterobacteriaceae (OTU 917; 8.22% and 2.99%) were found on Mw and BPSw, respectively, on day 0, but OTU 917 also appeared on days 7 and 14 in BPSwo (at 3.48% and 5.80% respectively). Other OTUs found in Mw on day 0 were a *Methylobacterium* sp. (OTU 34; 3.88%) and a *Sphingomonas* sp. (OTU 37; 3.98%). Similarly, in BPSw and BPSwo on day 0, a *Kosakonia* sp. (OTU 852; 6.42%) and Flavobacteriaceae (OTU 10; 10.59% and 4.2%) were found.

For the four types of fermentations, it can be observed that there was a distinct shift in the bacterial profiles between day 0 and day 7, and from then on there was a more gradual further evolution of the profile towards the end of the silage period. For Mwo (Fig. 2A), on day 7, there was a clear dominance of two *Lactobacillus* sp. (OTU 2; OTU 20), known as good lactic acid producers (Ni *et al*., 2017). However, this *Lactobacillus* species disappeared to some extent and dominance was taken over by day 90 by a *Buttiauxella* sp. (OTU 1; 10.5%), a *Weissella* sp. (OTU 6; 20.1%) and a number of other OTUs, each with an abundance below 3%. *Weissella* species are obligatory heterofermentative LAB species found in different forage crops. Their dominance can be seen as indication of a normal fermentation and it may be important in the aerobic stability of silage (Gharechahi *et al*., 2017). In Mw (Fig. 2B), a relatively similar evolution can be observed, with the difference that *Buttiauxella* (OTU 1) was present at a higher relative abundance on day 90 than in Mwo. The facultative anaerobic *Buttiauxella* species belong to the family of the Enterobacteriaceae. In the colony counts, no Enterobacteriaceae were detected as from day 7, but the DNA of *Buttiauxella* species was observed in the metagenetic analysis throughout the fermentation period. Either the detected DNA belonged to dead cells, or they were viable but not cultivable anymore (Andeta *et al*., 2018). The strictly aerobic bacteria that were present on day 0, i.e. *Methylobacterium* (OTU 34; 3.88%) and *Sphingomonas* (OTU 37; 3.98%) disappeared completely after day 0. For BPSwo (Fig. 3A) and BPSw (Fig. 3B), the dynamics are somewhat less evident, but for both types of silages, the abundances *Leuconostoc* (OTU 3) and *Buttiauxella* (OTU 1) faded away towards the end of the fermentation, while *Bifidobacterium* (OTU 4) clearly established a relative abundance of 28.5% and 24.5% at day 90 for BPSwo and BPSw respectively. In a study conducted by Li *et al*. (2019), in the evaluation of tropical forage grasses, *Bifidobacterium* was seen dominant with prolonged fermentation and increased temperature conditions. *Bifidobacterium* species are characterized by the conversion of free conjugate linoleic acid into conjugated linoleic acid, and they are known for their health benefits for both humans and animals (Coakley *et al*., 2003; Li *et al*., 2019). The dominance of *Bifidobacterium*, as seen in this study, may benefit ruminant animals (Li *et al*., 2019). The impact on the use of molasses on the bacterial profiles is more clear for banana pseudostem than for maize stover. By considering day 90 and disregarding the OTUs that do not belong to the category ‘other’, the OTUs belonging to the LAB are fairly similar for Mwo (32.2%, being the sum of 12.2% of OTU 282 and 20.1% of OTU 6) as for Mw (29%, being the sum of the abundances of OTUs 3, 282, 6, 2 and 20), in contrast to the clearly higher share of LAB for BPSw (sum of OTUs 973, 518, 8, 1138, 19, 2 and 3 is 53.7%) than for BPSwo (sum of OTUs 19 and 3 is 17.6%). This finding largely coincided with the LAB counts on day 90. Therefore, in the silage of banana pseudostem squeezed to reduce its moisture content, there is a need for molasses addition. Remarkably, OTUs related to *Clostridium* were only detected on day 30 and 60 in BPSwo. On these days, the *Clostridium* spore counts were also (slightly) higher for this treatment than for the other three treatments. It has to be noted, however, that as seen in previous research (Andeta et al., 2018, 2019), endospores are resilient to many traditional methods of DNA isolation and can therefore remain underdetected in metagenomics, even when a tailored DNA extraction method is used (Filippidou *et al*., 2015).

As shown in Table 2, the coverage, a parameter that indicates the percentage of individuals sampled in a microbial community, ranged from 60.4% to 80.5%. The observed richness indicates the number of OTUs observed in a sample, but the Chao1 index, estimates the true species diversity of a sample by also taking into account rare species (singletons and doubletons). The Chao1 index was higher than the observed richness. Based on these observations, it can be concluded that the bacterial community was not completely covered during sequencing. Nevertheless, it can be deduced that for maize stover, there was no clear trend when comparing Mwo and Mw, but for banana pseudostem, the observed richness of BPSwo was higher at any time in the fermentation than for BPSw. The higher diversity was likely due to the relatively higher pH values of BPSwo silages, which could not suppress the microbial growth to a substantial extent and subsequently reduce the bacterial community diversity (Ni *et al*., 2017). Therefore, the addition of molasses clearly affected the bacterial community into fewer but more dominant species in the silages of BPS. The Shannon–Wiener index describes diversity in terms of the observed richness combined with the relative abundance of the OTUs. The index increases as both richness and evenness of the community increase. For both crops, when molasses was used, this diversity index slightly decreased during the first 14 days and from then on it increased steadily. This phenomenon was not evident for any of the crops when no molasses was added.

**Table 2:** Diversity indices for samples obtained during fermentation of maize stover and banana pseudostem both without and with molasses for 90 days in microsilos and subjected to metagenetics analysis.^1^

Treatment	Sample day	Diversity index			
		Observed richness	Chao1^2^	Coverage (%)^3^	Shannon – Wiener^4^
Maize without molasses	0	111.50 ± 20.50^aC^	183.25 ± 12.37^aB^	60.60 ± 7.09b^A^	2.50 ± 0.07^aBC^
	7	179.50 ± 2.12^bBC^	265.18 ± 10.95^abAB^	67.73 ± 2.00^aA^	2.22 ± 0.02^bC^
	14	239.50 ± 19.09^aAB^	344.97 ± 32.23^aAB^	69.47 ± 0.96^aA^	2.55 ± 0.04^aBC^
	30	184.50 ± 37.47^aBC^	309.73 ± 92.02^aAB^	60.44 ± 5.86a^A^	2.44 ± 0.04^aBC^
	60	272.00 ± 7.07^aA^	442.10 ± 103.88^aA^	63.08 ± 13.22^aA^	2.77 ± 0.03^aAB^
	90	289.00 ± 9.10^aA^	349.81 ± 9.59^aAB^	82.68 ± 5.09^aA^	3.06 ± 0.19^aA^
Maize with molasses	0	410.50 ± 0.71^aA^	520.07 ± 11.55^aA^	78.95 ± 1.62^aA^	3.98 ± 0.04^aA^
	7	171.50 ± 3.54^bBC^	242.42 ± 39.65^abB^	71.58 ± 10.25^aA^	2.44 ± 0.03^bB^
	14	156.00 ± 1.41^aC^	235.43 ± 24.50^aB^	66.65 ± 7.54^aA^	2.17 ± 0.00^aB^
	30	216.50 ± 2.12^aBC^	337.02 ± 29.10^aAB^	64.51 ± 6.20^aA^	2.56 ± 0.01^aAB^
	60	226.00 ± 4.24^aBC^	304.08 ± 11.31^aB^	74.35 ± 1.40^aA^	2.85 ± 0.00^aAB^
	90	261.50 ± 64.35^aB^	362.16 ± 117.49^aAB^	73.17 ± 5.10^aA^	2.80 ± 0.91^aAB^
Banana pseudostem without molasses	0	259.50 ± 190.21^aA^	381.06 ± 264.30^aA^	66.87 ± 3.53^abA^	2.90 ± 0.89^aA^
	7	534.50 ± 173.24^aA^	667.02 ± 232.57^aA^	80.50 ± 2.09^aA^	4.59 ± 0.45^aA^
	14	285.50 ± 201.53^aA^	385.67 ± 253.90^aA^	72.55 ± 4.50^aA^	3.06 ± 0.90^aA^
	30	263.00 ± 224.86^aA^	359.04 ± 348.66^aA^	81.06 ± 16.09^aA^	3.32 ± 0.98^aA^
	60	231.00 ± 158.39^aA^	319.93 ± 214.02^aA^	71.68 ± 1.56^aA^	3.25 ± 0.44^aA^
	90	200.00 ± 135.76^aA^	244.18 ± 139.79^aA^	78.92 ± 10.41^aA^	2.73 ± 0.65^aA^
Banana pseudostem with molasses	0	114.50 ± 4.95^aA^	167.42 ± 9.93^aA^	68.42 ± 1.10^abA^	2.39 ± 0.00^aB^
	7	93.00 ± 2.83^bB^	143.50 ± 19.09^bA^	65.25 ± 6.71^aA^	2.21 ± 0.01^bC^
	14	101.00 ± 0.00^aAB^	147.54 ± 21.87^aA^	69.22 ± 10.26^aA^	2.07 ± 0.04^aD^
	30	93.00 ± 1.41^aB^	160.63 ± 19.11^aA^	58.26 ± 6.02^aA^	2.21 ± 0.00^aC^
	60	108.00 ± 5.66^aAB^	142.88 ± 17.20^aA^	75.90 ± 5.18^aA^	2.44 ± 0.01^aB^
	90	105.00 ± 9.89^aAB^	148.23 ± 21.25^aA^	71.09 ± 3.51^aA^	2.64 ± 0.04^aA^

^a,b,c^Different superscripts within the same column and within the same treatment for the different sampling days indicate significant differences (*P* < 0.05).

^A,B,C^Different superscripts within different treatments, but same sampling day, indicate significant differences (*P* < 0.05).

^1^ Data are the mean values of two sequenced extracts ± standard deviations.

^2^ Chao1 index: the total number of OTUs estimated for infinite sampling (Chao, 1984).

^3^ Coverage was determined by using the equation (observed richness/Chao1 estimate) × 100.

^4^ Shannon–Wiener index: index to characterize species diversity based on species richness as well as their relative abundance. A higher value means more diversity (Shannon, 1948). Data among sampling days and sample types were analysed using one‐way analysis of variance (ANOVA). The statistical differences among means having significant difference were determined using *Turkey’s* multiple comparisons. Differences were considering significance at *P* < 0.05.

## Conclusions

It can be concluded from this study that both maize stover and banana pseudostem, when ensiled under conditions prevailing in South Ethiopia, show a microbial profile during as generally expected for ensiled crops, but in the case of banana pseudostem, squeezing or pressing as well as the addition of molasses are necessary before the fermentation. Except for banana pseudostem without molasses, the pH and the microbiota during fermentation showed dynamics that are known to characterize proper silage processes. The culture‐dependent counts and the metagenetic analyses revealed corresponding findings, but at the same time provided complementary information. The dominance of *Bifidobacterium* observed in the last days of the silages of banana origin may have additional health benefits for ruminants. Further research should focus on the chemical and nutritional quality of the silages as well as on the opportunity to ferment mixtures of the two crop by‐products.

## Experimental procedures

### Forage harvest and silage preparation

Prior to collecting biomass for fermentation, 60 microsilos were constructed with a capacity of 4 l (10 cm diameter and 50 cm height) using unplasticized polyvinyl chloride (uPVC) tubes. The microsilos were sealed at the bottom using gas tight glue (PVC glue, China), checked for water leakage and disinfected using 98% ethanol.

Maize (*Zea Mays*, BH‐140) was grown at the Arba Minch University farm, which is located at 437 km south of Addis Ababa in the southern hot humid rift valley region of Ethiopia. The spikes of the maize at milk‐line kernel maturity stages were harvested for human use (Israelsen *et al*., 2009; Gharechahi *et al*., 2017). The maize stover was immediately harvested manually at about 10 cm height and chopped using a forage chopper (Electro Mecce, Engineering Service, Ethiopia) to an average particle length of 2 to 4 cm. In this way, a total of 200 kg of maize stover were obtained, homogenized and equally divided into two 100 kg aliquots. One aliquot was divided over a series of 15 microsilos, and as no molasses was added, this treatment is further referred to as maize without molasses (Mwo). The other half (100 kg) was mixed thoroughly with 5% (w/w) molasses and divided over another series of 15 microsilos, referred to as maize with molasses (Mw). In the current study, molasses with 16.2% DM content was added to compensate for the loss of WSC by removing the spikes (grain) from the maize or by squeezing the banana pseudostem to increase its DM content. The filling of the microsilos was performed using hand tools, and the biomass was compressed to 80% of the microsilo volume. Compression forced out the trapped oxygen to reduce further plant respiration and to obtain anaerobic conditions (McEniry *et al*., 2008; Gharechahi *et al*., 2017). In each microsilo, a cement stone designed to tightly fit within the microsilo and weighing 1.5 kg was placed on the top of the biomass to maintain pressure before the microsilo was sealed with a PVC cap and scotch tape (Wang *et al*., 2016; Andeta *et al*., 2018). Subsequently, the top caps were equipped with a CO_2_ valve.

Banana pseudostem (*Musa acuminata*, BPS) was randomly collected from a local farmer’s field in the neighbourhood of the Arba Minch University after the banana fruits were harvested. The BPS was cut into short pieces using big knife and chopped to an average length of 2–4 cm by using the same chopping machine mentioned above. Then, the chopped banana pseudostem products were squeezed manually to reduce the moisture content following the procedure stated by Andeta *et al*. (2018), in enset (*Ensete ventricosum*) fermentation. A total amount of 200 kg chopped and squeezed BPS was obtained and divided into two aliquots of 100 kg. One aliquot was divided over a series of 15 microsilos, and as no molasses was added, this treatment is further referred to as BPS without molasses (BPSwo). The other half was mixed thoroughly with 5% molasses (w/w) and divided over another series of 15 microsilos, referred to as BPS with molasses (BPSw). The microsilos were compacted and closed in the same manner as described for maize. All microsilos were stored in the same laboratory room until their respective sampling dates.

### Sampling

On day 0 prior to ensiling, fresh samples were taken and stored under refrigerated conditions for maximum 24 h until analyses of the microbial population of the raw material was performed. After 7, 14, 30, 60 and 90 days silage, triplicate microsilos were destructively sampled per treatments as described by McEniry *et al*. (2008) and Andeta *et al*. (2018). Samples of about 80 g were taken at a depth of about 20–30 cm of each microsilo in a sterile way and immediately subjected for the DM, pH and microbial count analyses explained in the next sections. 15 g of each of these samples was freeze‐dried for 24 h at −50°C (Alpha 1‐4 LD plus freeze‐drier, Martin Christ, Osterode am Harz, Germany) and stored at −18°C for a period of 4 months before metagenetic analyses.

### Dry matter content and pH

The dry matter content and pH were measured according to the methods used by Moselhy *et al*. (2015) and Andeta *et al*. (2018) using the oven‐drying method and a digital pH meter (PH 1100H, VWR International, Darmstadt, Germany) respectively. The average and standard deviation were calculated for the three microsilos per treatment.

### Microbial counts

Classic colony counts were determined as described by Andeta *et al*. (2018) and McEniry *et al*. (2008). Five grams of sample was transferred aseptically into a sterile stomacher bag, and 45 ml of peptone physiological salt solution (0.85% NaCl, 0.1% peptone, Biokar Diagnostics, Beauvais, France) was added. The mixture was homogenized for 60 s (StarBlender™ LB 400, VWR International, Fontenay Sous Bois Cedex, France). A ten‐fold serial dilution was plated on different media using the pour‐plate method. Total viable aerobic counts were determined on Plate Count Agar incubated at 30°C for 3 days, Enterobacteriaceae on Violet Red Bile Glucose Agar incubated at 37°C for 24 h, LAB on de Man Rogosa Sharpe Agar incubated at 30°C for 3 days, yeasts and moulds on Dichloran Glycerol (DG‐18) agar supplemented with 0.1 g l^−1^ chloramphenicol, incubated at 25°C for 5 days. *Clostridium* endospores were counted by giving the ten‐fold serial dilution a heat shock treatment (10 min at 80°C), followed by serial dilution, plating onto Reinforced *Clostridium* Agar (RCA) and anaerobic incubation at 37°C for 48 h using anaerobic jars, gas generating kits and indicator strips (IVD, Microbiology Anaerotest^®^, Merck KGaA, Darmstadt, Germany). As a heat shock treatment was included in the counting protocol for *Clostridium*, only *Clostridium* spores were counted rather than a total *Clostridium* count including also the vegetative cells. This approach was used because the medium available, RCA, also supports the growth of lactobacilli and other anaerobes. By combining it with a heat treatment and anaerobic incubation, *Clostridium* could be determined specifically. All media were obtained from Biokar Diagnostics (Beauvais, France). Microbial counts were determined for each replicate and expressed as log cfu g^−1^.

### Bacterial community composition by metagenetics

Five grams of freeze‐dried silage samples was aseptically crushed and homogenized in 45 ml of sterile peptone water (peptone, 1 g l^−1^) for 2 min at 260 rpm using a Bagmixer^®^ Stomacher (Interscience, Saint Nom, France). The homogenate was centrifuged at 16 000 *g* for 5 min. The total bacterial DNA was extracted from 0.25 g of pellet using the Power soil DNA Isolation Kit (Mo Bio Laboratories, Carlsbad, California, USA) following the manufacturer’s instruction. Afterwards, replicate DNA extracts were pooled and diluted 10 times. Subsequently, PCR amplification, library preparation, sequencing, sequence processing and diversity analyses were performed as described by Andeta *et al*. (2019). Observed richness, Chao1, Coverage and Shannon–Wiener diversity indices were calculated using the R package Phyloseq v1.16.2 (R‐package, R Development Core Team 2013).

### Statistical analysis

Differences in the dry matter content, pH, microbial counts and diversity indices (richness, Chao1, Coverage and Shannon–Wiener diversity index) throughout the different sampling days and between the four sample types were analysed using one‐way analysis of variance (ANOVA). All tests were performed using SPSS (IBM©SPSS Statistics v. 23, New York, USA). For treatment means showing significant differences, multiple comparisons were performed using Turkey’s HSD, while considering a significance level of 0.05.

## Conflict of interest

The authors declare that there is no conflict of interest.
